# Neutrophils in primary gastric tumors are correlated with neutrophil infiltration in tumor-draining lymph nodes and the systemic inflammatory response

**DOI:** 10.1186/s12865-018-0251-2

**Published:** 2018-04-16

**Authors:** Soichiro Hiramatsu, Hiroaki Tanaka, Junya Nishimura, Chie Sakimura, Tatsuro Tamura, Takahiro Toyokawa, Kazuya Muguruma, Masakazu Yashiro, Kosei Hirakawa, Masaichi Ohira

**Affiliations:** Department of Surgical Oncology, Osaka City University Graduate School of Medicine, 1-4-3 Asahi-machi, Abeno-ku, Osaka, 545-8585 Japan

**Keywords:** Tumour-associated neutrophils, Gastric cancer, Prognosis, Draining lymph node

## Abstract

**Background:**

Tumor-Associated Neutrophils (TANs) may be able to induce lymphangiogenesis and angiogenesis, although the detailed roles of TANs remain unclear. The Neutrophil-Lymphocyte Ratio (NLR) is an inflammation-based prognostic factor for gastric cancer. This study aimed to investigate the distribution of CD15^+^neutrophils in the primary tumor and Tumor-Draining Lymph Nodes (TDLNs), and to examine the association of TANs with the clinicopathological features (including NLR) of patients with gastric cancer.

**Results:**

Immunohistochemical staining showed that the median number of CD15^+^TANs was 18 and 24 per high-power field (HPF) in primary tumors and TDLNs, respectively. Patients were divided into high and low infiltration groups based on the median number. A high number of infiltrating CD15^+^TANs in the primary tumors and in the TDLNs were associated with depth of invasion and lymph node metastasis. Kaplan-Meier analysis revealed that a poor overall survival was associated with high numbers of CD15^+^TANs, and the multivariate analyses revealed that a high number of CD15^+^TANs in the TDLNs was an independent prognostic factor. The numbers of CD15^+^TANs in the primary tumors and TDLNs showed weak positive correlation. The number of CD15^+^TANs in the primary tumors was positively correlated with the preoperative NLR, (*P* = 0.001, *R* = 0.327) and immunohistochemical staining revealed that C-X-C motif chemokine receptor 2 (CXCR2) ^+^neutrophils might be the origin of the CD15^+^TANs. Flow cytometry analysis indicated that infiltrating neutrophils increased in the tumor and TDLN compared to non-cancerous tissue. Neutrophils treated with cancer supernatant upregulated TWIST and IL-6 genes in vitro.

**Conclusion:**

Our findings suggested that local infiltration of CD15^+^TANs may be correlated with inflammation in TDLNs and systemic response to cause metastasis in gastric carcinoma.

## Background

Advanced gastric cancer has a poor prognosis, despite the development of novel treatments, and lymph node metastasis is an important prognostic factor in cases of gastric cancer [1, 2]. We have previously reported that lymphangiogenesis was augmented in both of the primary gastric carcinoma and the tumour-draining lymph nodes (TDLNs) [3–5]. Furthermore, chronic inflammation involves immune cell infiltration, fibroblast proliferation, and angiogenesis, which are also observed in the tumor microenvironment [6, 7]. Moreover, intratumoral infiltration of immune cells is associated tumor invasion and metastasis [7–9]. Neutrophils are essential effector cells in the host’s defence against invasive pathogens, and could promote tumor progression through angiogenesis, lymphangiogenesis, and immune suppression as Tumor-associated neutrophils (TANs) [3, 10–13]. Although regional lymph nodes are considered an immune barrier for cancer, the detail mechanisms of lymph node metastasis remain unclear. Nevertheless, systemic inflammatory markers, including the Neutrophil-lymphocyte ratio (NLR), are prognostic factors in several cancers [13–15]. CD15, a carbohydrate adhesion molecule, is commonly used marker to identify human neutrophils and is known to mediate chemotaxis and phagocytosis. It has also been reported that some type of granulocytic myeloid-derived suppressor cells (MDSCs) which have immunosuppressive functions express CD15 [16]. Thus, CD15 is one of the effective molecules for searching the immunological function of neutrophils in the tumor microenvironment. Therefore, the present study aimed to explore the relationships of CD15^+^TANs with gastric cancer and systemic inflammation, based on the microenvironments in the primary tumor and TDLNs.

## Methods

### Patients and surgical specimens

We retrospectively examined surgical specimens from patients who underwent gastrectomy at our department during 2007–2008. The specimens were formalin-fixed and paraffin-embedded tissues from 120 primary tumours and 497 TDLNs, which were analysed using immunohistochemistry. For the present study, TDLNs were defined as 4–5 lymph nodes near the primary tumor. Pathological staging was performed according to the seventh edition of the International Union Against Cancer Tumour-Node-Metastasis classification. Postoperative follow-ups were performed every 3 months for the first 2 years, and then every 6 months during years 3–5.

### Immunohistochemical staining

Sections with a thickness of 4 μm were obtained from the paraffin-embedded blocks. After incubation at 60 °C for 10 min, the sections were deparaffinized using xylene and rehydrated using a graded series of ethanol. The slides were subsequently washed twice for 5 min in phosphate-buffered saline (PBS). Endogenous peroxidase activity was blocked for 15 min using absolute methanol containing 3% hydrogen peroxide. After washing the sections in PBS, they were microwaved for 10 min to achieve antigen retrieval. Non-specific binding was blocked using a non-specific staining blocking reagent (Dako, Kyoto, Japan). The sections were then incubated overnight at 4 °C with mouse monoclonal antibodies (CD15 or C-X-C motif chemokine receptor 2 (CXCR2), 1:100 dilution; Abcam, Tokyo, Japan), and subsequently washed using PBS for 10 min before a 10-min incubation with a secondary antibody at room temperature. After washing the sections using PBS, they were visualized using 3-3′-diaminobenzidine for 5 min and then counter-stained using haematoxylin before mounting.

### Markers of systemic inflammation

The NLR was retrospectively calculated based on routine test results from peripheral blood samples that were collected within 2 weeks before the operation. In cases with multiple blood samples, the sample from the first hospital visit was used to calculate the NLR. The median preoperative NLR value (1.932) was used as a cut-off to classify patients as having a high pre-operative NLR (≥1.932) or a low pre-operative NLR (< 1.932). The numbers of TANs were compared between the high and low preoperative NLR groups.

### Flow cytometry

Whole single-cell suspensions collected from tumor, non-tumor region lymph nodes, and TDLNs were incubated with CD16b-PE antibodies (BD Pharmingen), and then analyzed by flow cytometry. Flow cytometric analyses were performed using a BD LSR II flow cytometer with FACSDiva™ software (both from Becton-Dickinson).

### Neutrophils isolation and culture

Human neutrophils were isolated from healthy donors using Polymorphprep (Axis- Shield) centrifugation (purity≧85%). Cells were washed three times in complete RPMI 1640 (Sigma-Aldrich) (with 100 U/ml penicillin, 100 μg/ml streptomycin, and L-glutamine (Hyclone), and 10% fetal bovine serum (Gibco)). Then, neutrophils were incubated in complete RPMI 1640 at 37 °C and 5% CO2 for 16 h. OCUM12 were maintained in DMEM (Wako, Osaka, Japan) supplemented with 10% fetal bovine serum (FBS; Nichirei Bioscience, Tokyo, Japan) and 20% penicillin-streptomycin (Wako, Osaka, Japan). OCUM12 cell lines derived from human gastric cancer were suspended at a density of 1 × 10^5^ per ml in RPMI1640 were incubated for 24 h. Then we stimulated neutrophils from healthy donors using 50% supernatant of OCUM12 for 24 h. We defined neutrophils stimulated with the supernatant of OCUM12 as TAN in this experiment.

### Tumor-neutrophil co-culture system

We seeded 1 × 10^5^ OCUM12 cells in 500 μl DMEM on 24-well plates, and neutrophils or TAN adjusted to 1 × 10^5^ cells per 500 μl DMEM were co-cultured with OCUM12 using cell culture Inserts (BD, Falcon) at 37 °C and 5% CO2 for 16 h. After 16 h, OCUM12 was removed from medium, centrifuged, and washed three times by PBS. RNA was then extracted from the isolated OCUM12 cells.

### RNA extraction and reverse transcription reactions

RNA easy Mini Kits (QIAGEN, Tokyo, Japan) were used according to the manufacturer’s instructions to extract total RNA. ReverTra Ace qPCR RT Master Mix (TOYOBO, Osaka, Japan) was used according to the manufacturer’s instructions to reverse transcribe the isolated RNA and generate single-strand cDNA; this reaction was performed at 37 °C for 15 min, 50 °C for 5 min, and 98 °C for 5 min. 

### Quantitative reverse-transcription PCR

We analyzed the expression of mRNA of interleukin 6 (IL-6) in neutrophils. We also searched the expression of TWIST in OCUM12 via qRT-PCR. TaqMan PCR core reagents were used for these assays; each reaction was recorded and analysed with the ABI PRISM 7000 Sequence Detection System. After an initial denaturation for 10 min at 95 °C, each sample was subjected to 40 cycles of PCR (95 °C for 15 s, and 60 °C for 1 min, per cycle). To analyze the ratios of gene transcription levels, glyceraldehyde 3-phosphate dehydrogenase (GAPDH) was used as an internal control. The 2^−ΔΔCt^ method was used to determine the relative expression of each target gene. All experiments were performed in triplicate, and mean values were used for further calculation.

### Informed consent

This study’s retrospective protocol was approved by the Osaka City University ethics committee, and informed consent was obtained in writing from all patients and participants for collection and analysis of the specimens in this study.

### Statistical analysis

Continuous variables were compared using Student’s t test, and categorical variables were compared used the chi-square test. Overall survival curves were calculated using the Kaplan-Meier method and differences were evaluated using the log-rank test. Prognostic factors were examined using univariate and multivariate analyses with a Cox proportional regression model. Differences were considered statistically significant at *P*-values of < 0.05. All statistical analyses were performed using JMP software (version 11; SAS Institute, Cary, NC, USA).

## Results

### Correlations of tumour-infiltrating CD15^+^TANs with clinicopathological features

**Fig. 1:**
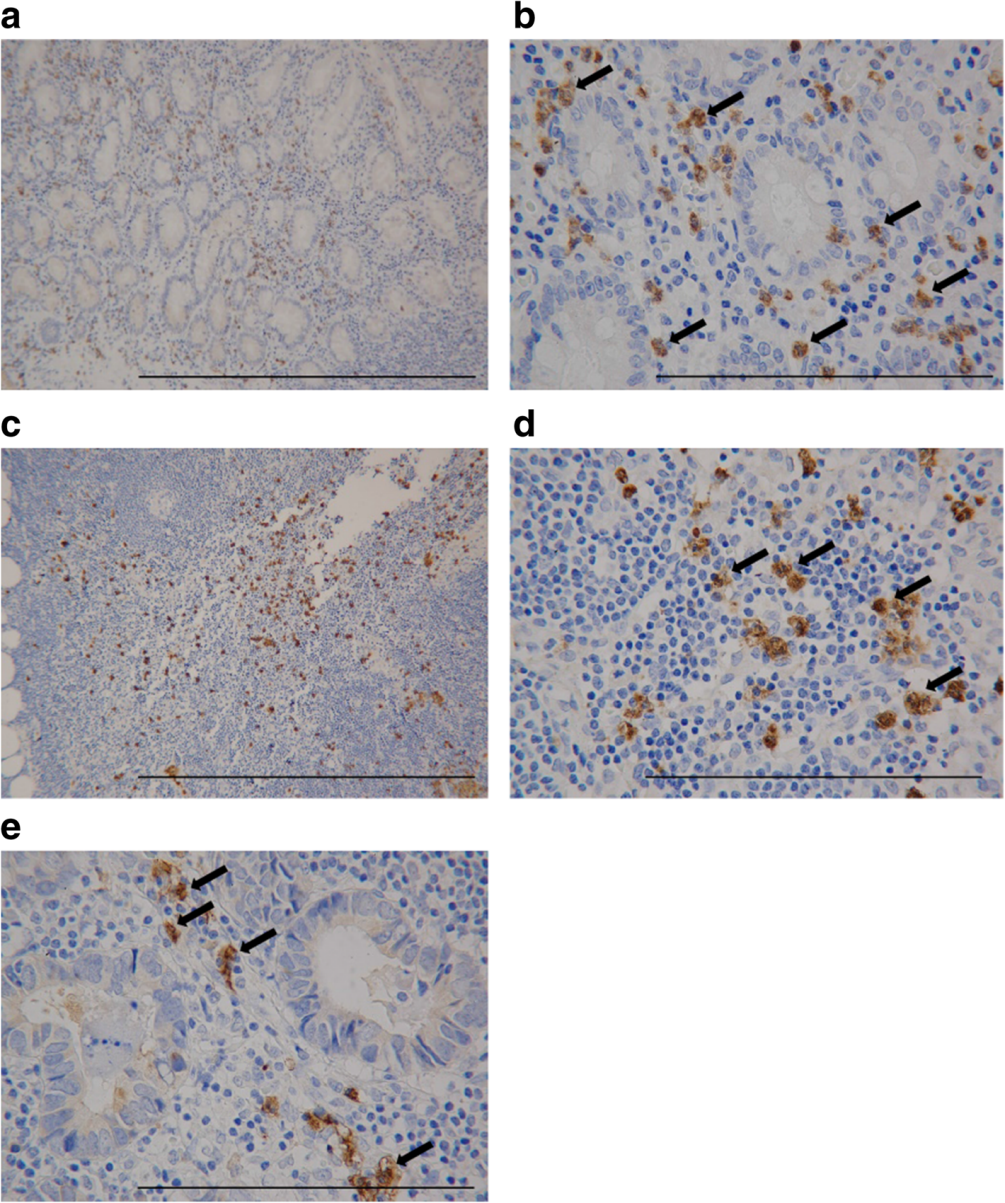
Immunohistochemical staining of CD15^+^Tumor-associated Neutrophils (TANs) of patients with gastric cancer. CD15^+^TANs are identified as brown-stained segmented cells (*arrows)*. CD15^+^TANs were mainly stained on the membrane and perinuclear. (× 400) *Scale bar* 0.1 mm. **a**: High expression of CD15^+^TANs in primary tumor (× 40) *Scale bar* 1 mm*.* **b**: CD15^+^TANs in primary tumor infiltrated to surround tumor cells. (× 400) *Scale bar* 0.1 mm. **c**: High expression of CD15^+^TANs in lymph nodes (**b**) (× 40) *Scale bar* 1 mm. **d**, **e**: Distribution of CD15^+^TANs in lymph nodes (× 400). **d**: diffusely area. E: peri-metastatic cell

CD15^+^TANs were identified based on brown membrane and perinuclear staining, and were observed to have infiltrated the primary tumour and surrounded the tumour cells (Fig. 1a, b). On the other hand, CD15^+^TANs in TDLNs were observed around metastatic cells (Fig. 1e), or diffusely areas (Fig. 1c, d).

Patients were divided into high and low TAN infiltration groups, based on median CD15^+^TAN number of 18.4 per high-power field in primary tumours and 23.9 per high-powered field in lymph node.

**Fig. 2:**
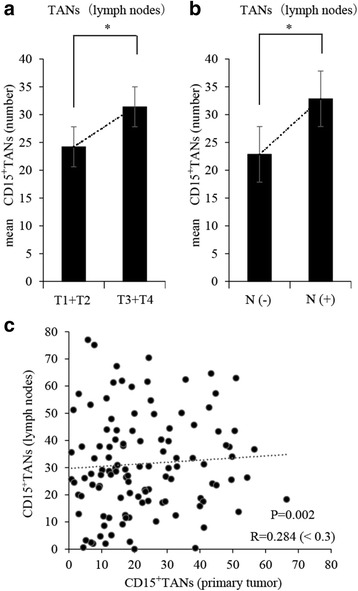
Correlation between CD15^+^TANs in lymph nodes and clinicopathological features of patients with gastric cancer, or CD15^+^TANs in primary tumor. Based on the median number of CD15^+^TAN in lymph nodes (23.9 cells/HPF), the patients were divided into high and low CD15^+^TANs groups. **a**, **b**: The number of CD15^+^TANs in lymph nodes were significantly associated with pT3/4 disease (*P* = 0.036) and lymph node metastasis. (*P* = 0.002). **c**: A scatter chart of the number of TANs in primary tumor and in lymph nodes. Weak positive correlation was showed between TANs in primary tumor and in lymph nodes. (P = 0.002 *R* = 0.284) *p < 0.05

**Fig. 3:**
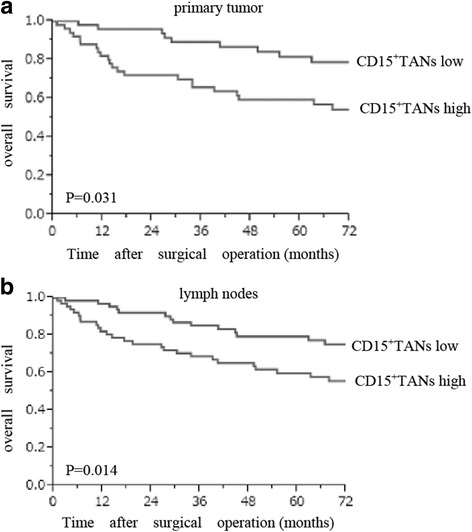
The Kaplan-Meier survival analysis of CD15^+^TANs in primary tumor and in lymph nodes. **a**: primary tumor **b**: lymph nodes. Based on the median number of CD15^+^TAN (primary tumors 18.4 cells/HPF lymph nodes 23.9 cells/HPF), the patients were divided into high and low CD15^+^TANs groups. Log-rank test shows that both primary tumor and lymph nodes, patients with high CD15^+^TANs group were poorer prognosis than those with low CD15^+^TANs group. (primary tumor: *P* = 0.031, lymph nodes: *P* = 0.014)

High infiltration of CD15^+^TANs in the primary tumor was associated with pT3/4 disease, lymph node metastasis, and positive vessel involvement (Table 1). Patients with pT3/4 disease and lymph node metastasis had high numbers of CD15^+^TANs (Fig. 2a, b). A scatter chart of CD15^+^TANs in the primary tumor and TDLNs revealed a weak positive correlation (*P* = 0.002, *r* = 0.284) (Fig. 2c). The Kaplan-Meier survival analysis of CD15^+^TANs showed that both primary tumor and lymph nodes, patients with high CD15^+^TANs group were poorer prognosis than those with low CD15^+^TANs group. (primary tumor: *P* = 0.031, lymph node: *P* = 0.014) (Fig. 3a, b). In addition, overall survival was univariately associated with depth of invasion, lymph node metastasis, International Union Against Cancer stage, histological type, lymphatic invasion, and a high number of CD15^+^TANs in the primary tumour and in the TDLNs (Table 2). Multivariate analysis revealed that the independent prognostic factors were undifferentiated type, a pathological stage of III/IV, and high CD15^+^TAN infiltration in TDLN (Table 2).

**Table 1:** Relationship between CD15 + TANs of primary tumour and clinicopathological features of patients with gastric cancer

characteristics	N	TAN(primary tumour)		*P* value
		high	low	
Age(years)				
< 65	56	27	29	0.987
≧65	64	31	33	
gender				
Male	87	39	48	0.023
female	33	20	13	
pT category				
T1 + T2	69	24	45	< 0.001
T3 + T4	51	34	17	
pN category				
N(−)	67	21	46	< 0.001
N(+)	53	38	15	
pStage				
I + II	78	27	51	< 0.001
III + IV	42	31	11	
Histology				
differentiated(well, mod, pap)	52	23	29	0.1810
undifferentiated(por, sig, muc)	68	36	32	
lymphatic invasion				
absent	48	15	33	< 0.001
present	72	43	29	
venous invasion				
absent	91	39	52	0.023
present	29	10	19	

*P value < 0.05*

**Table 2:** Univariate and multivariate analysis of overall survival of patients with gastric cancer

factor	Univariate analysis	*P* value	Multivaliate analysis	*P* value
	HR(95%CI)		HR(95%CI)	
histology				
undifferentiated vs differentiated	1.805(1.138-2.933)	0.011	2.155(1.084-4.540)	0.028
pT Stage				
III + IV vs I + II	5.546(3.334-9.633)	< 0.001	–	–
pN Stage				
(+) vs (−)	4.129(2.500-7.092)	< 0.001	–	–
pStage				
III + IVvsI+II	5.124(3.193-8.355)	< 0.001	3.116(1.330-7.977)	0.008
lymphatic invasion				
present vs absent	3.882(2.228-7.251)	< 0.001	0.594(0.151-2.218)	0.443
venous invasion				
present vs absent	1.469(0.836-2.460)	0.173	–	–
CD15 + TANs (primary)				
high vs low	2.157(1.081-4.588)	0.028	1.391(0.676-3.038)	0.376
CD15 + TANs (lymph)				
high vs low	2.150(1.122-4.278)	0.013	1.997(1.036-4.051)	0.038

*HR* Hazard ratio, *Cl* confidence interval. *P value < 0.05*

### Expression of CXCR2 in the TANs

**Fig. 4:**
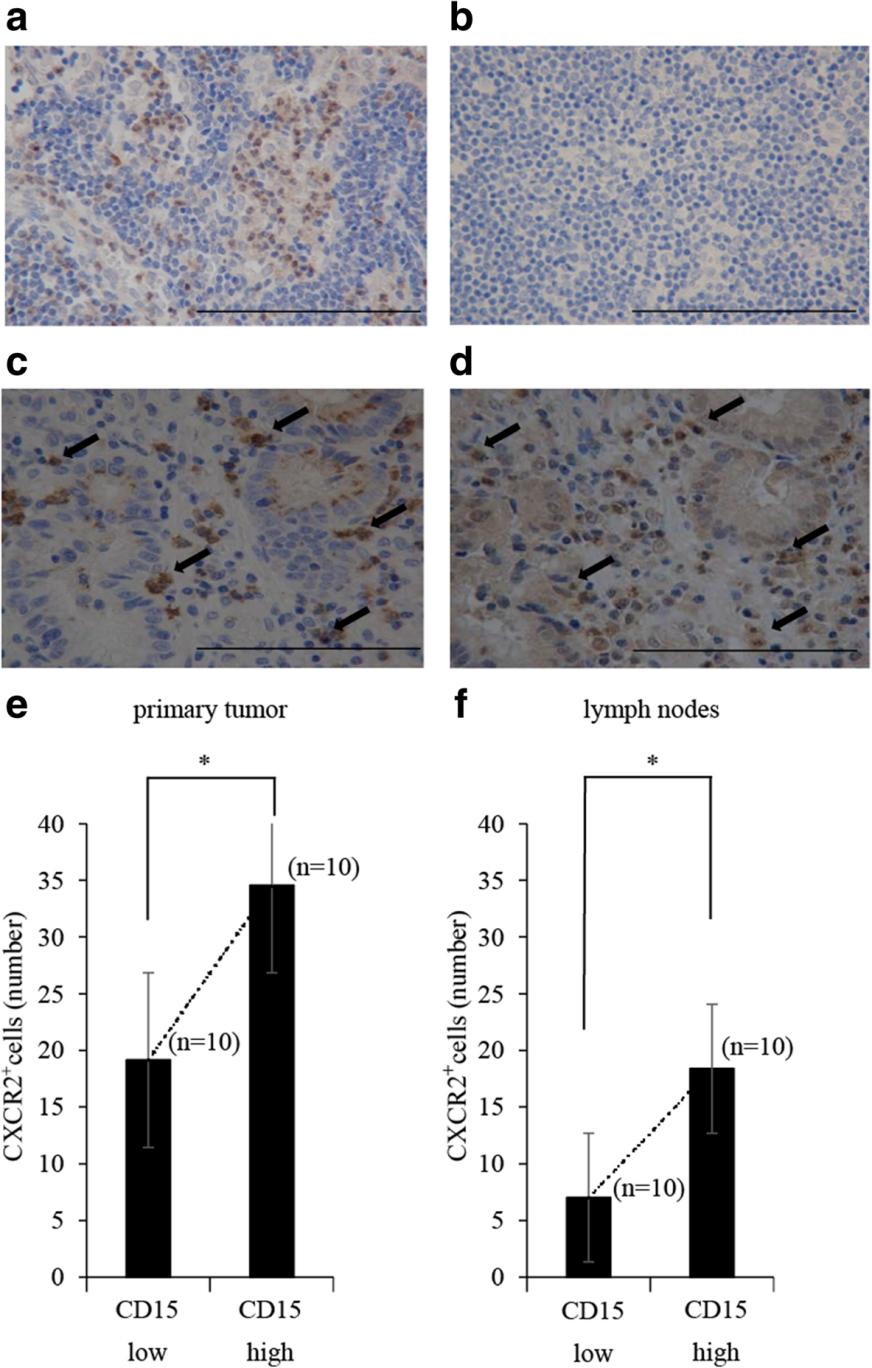
Immunohistochemical staining of CXCR2^+^neutrophils with anti-CXCR2 antibody (Abcam, Tokyo, Japan) in primary tumor and lymph nodes. **a**: High expression of CXCR2^+^neutrophils in lymph node. (× 400) *Scale bar* 0.1 mm. **b**: Low expression of CXCR2^+^neutrophils in lymph node. (× 400) *Scale bar* 0.1 mm. **c**,**d**: The same section of primary tumors were immunostained with anti-CD15 antibody and anti-CXCR2 antibody, and its positional relationship was examined. CD15^+^TAN and CXCR2^+^neutrophis were similarly expressed at the position. **c**: Infiltration of CD15^+^TANs around tumor cell in primary tumor. (× 400) *Scale bar* 0.1 mm D: CXCR2^+^neutrophils infiltrated into the same position as CD15 + TAN. (× 400) *Scale bar* 0.1 mm **e**, **f**: Comparative study on the number of CXCR2^+^neutrophils in each group. The number of CXCR2^+^neutorophils were significantly higher in high CD15^+^TANs groups than low CD15^+^TANs groups in both primary tumor and lymph nodes. (primary tumor: *P* < 0.001, lymph node: *P* < 0.001) *p < 0.05

We performed immunohistochemistry to detect CXCR2 expression to determine the origin of the TANs. CXCR2^+^neutrophils were identified as brown-stained segmented cells that surrounded the tumour cells (Fig. 4d). We also observed that CXCR2^+^ neutrophils and CD15^+^TANs exhibited similar infiltration locations in the same section of primary tumors (Fig. 4c, d). In both the primary tumors and the TDLNs, the expressions of CXCR2 were significantly elevated in the high CD15^+^TAN group, compared to the low CD15^+^TAN group (Fig. 4e, f).

### Correlation of TANs with the systemic inflammatory response

**Fig. 5:**
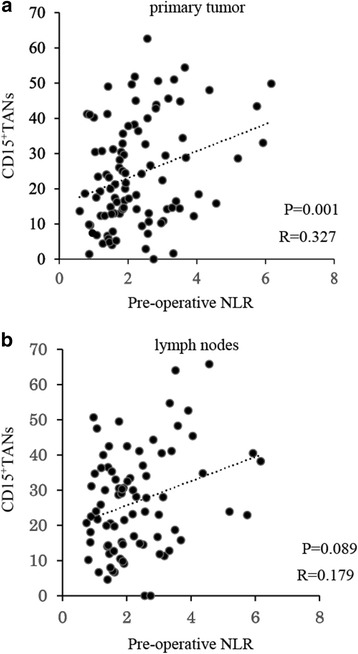
**a**, **b** The scatter chart of CD15^+^TANs and pre-operative Neutrophil-to-Lymphocyte Ratio (NLR). **a**: In primary tumor, Positive correlation was observed between CD15^+^TANs and pre-operative NLR. (*P* = 0.001 *R* = 0.327). **b**: There have no correlation between CD15^+^TANs in lymph nodes and pre-operative NLR. (*P* = 0.089 *R* = 0.179)

**Fig. 6:**
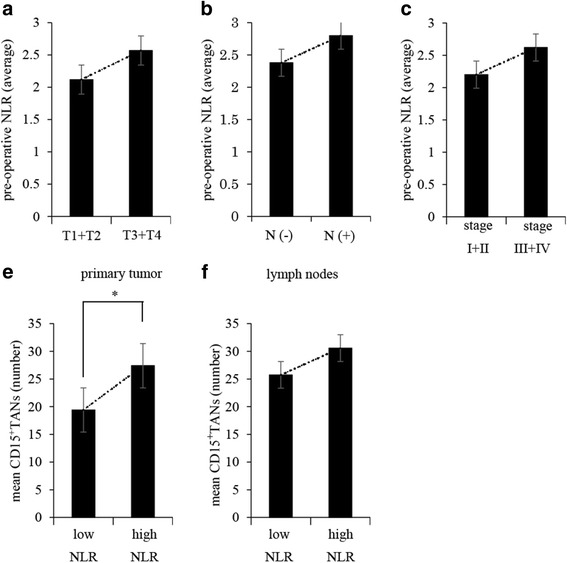
Correlation between pre-operative NLR of patients with gastric cancer and CD15^+^TANs in gastric cancer tissue. **a**, **b**, **c**: Relationship between pre-operative NLR and tumor progression. **a**: pT3/T4 disease **b**: lymph nodes metastasis **c**: pStage. **e**, **f**: Comparative study on the number of CD15^+^TANs in each group. The patients were divided into two groups based on the median pre-operative NLR value. (1.93). The number of CD15^+^TANs in primary tumors were significantly high in the high NLR group. (*P* > 0.001)

We also explored the correlation of intratumoral TANs with the pre-operative NLR, as a marker of the systemic inflammatory response. A scatter chart of CD15^+^TANs in the primary tumor and the pre-operative NLR revealed a positive correlation (Fig. 5a, P = 0.001, *r* = 0.327). Furthermore, the average NLR value increased with tumor progression (Fig. 6a–c). The patients were subsequently divided into two groups based on the median pre-operative NLR value (1.93, range: 0–18.4). Patients with a high NLR had a significantly higher average number of CD15^+^TANs in the primary tumors, compared to patients with a low NLR (Fig. 6d).

### The effect of TANs on inflammation and metastasis

**Fig. 7:**
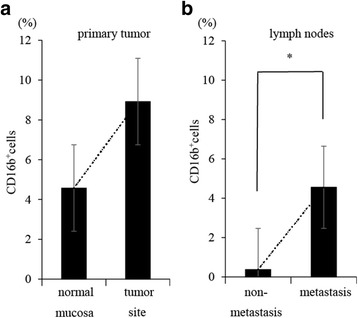
Comparison of neutrophils infiltration between tumor area and normal area. Lymph nodes, primary tumors, and normal mucosa were collected from patients who underwent surgical resection for gastric cancer. We used these samples for making single cell suspensions, then analyzed the ratio of neutrophils in each samples by flow cytometry using anti-CD 16b antibody. (BD Biosciences, San Jose, CA, USA). **a**: Comparison between tumor area and normal mucosa. **b**: Comparison between metastasis lymph nodes and non-metastasis lymph nodes

**Fig. 8:**
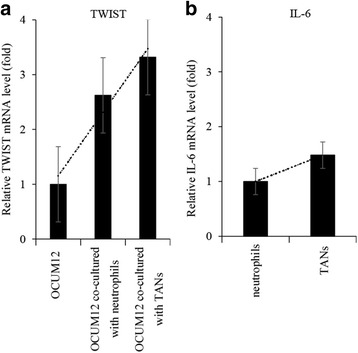
Alteration in mRNA expression in OCUM12 co-cultured with TANs and induction of inflammation by TANs. We investigated the expression of TWIST in OCUM12 co-cultured with TANs by PCR arrays. We also compared the expression of mRNA coding IL-6 in TAN and normal neutrophils. We defined neutrophils stimulated with the 50% supernatant of OCUM12 as TAN in this experiment. **a**: TANs induced increases in expression of mRNAs encoding TWIST in OCUM12. **b**: Comparison of expression of IL-6 in TAN and normal neutrophils

Our results suggested that TAN may contribute to local inflammation and induction of metastasis. Therefore, we investigated a mechanistic link between TAN and inflammation or metastasis by in vitro experiment. Flow cytometry showed that the ratio of neutrophils in gastric cancer tended to be higher than that of normal mucosa. (*P* = 0.057) (Fig. 7a). In addition, neutrophils in metastatic lymph nodes were significantly more than lymph nodes without metastasis. (*P* = 0.016) (Fig. 7b) To determine the effects of TANs on cancer cells, we also examined the expression of mRNA encoding Epithelial-Mesenchymal Transition (EMT) associated proteins (TWIST) in gastric cancer cell line, OCUM12. qRT-PCR showed that mRNA encoding TWIST were upregulated in OCUM12 co-cultured with TANs (3.32-fold). (Fig. 8a) Next, to examine the influence of TAN on inflammation, we compared the expression of mRNA coding IL-6 in neutrophils and TANs. The expression of mRNA encoding IL-6 in TANs were upregulated than that of neutrophils (1.48-fold) (Fig. 8b).

## Discussion

The present study revealed that the number of TANs in the primary tumors was associated with tumor progression and an increased number of TANs in the TDLNs. Furthermore, we observed that high TAN infiltration was correlated with the pre-operative NLR, which is a marker of systemic inflammation. CXCR2^+^neutrophils and CD15^+^TANs expressed similar distribution in the primary tumors. In addition, TANs had higher expression of IL-6 than normal neutrophils, and be increased the expression of TWIST which is known as one of the EMT marker. Our findings suggest that tumor-infiltrating neutrophils might be related to metastasis of gastric cancer that is caused by inflammation.

Neutrophils are the most abundant white blood cells and reportedly contribute to tumour progression [17–19]. Moreover, neutrophils release several factors that can stimulate tumorigenesis and angiogenesis, such as vascular endothelial growth factor (VEGF), Matrix metalloproteinase9 (MMP9), Myeloperoxidase (MPO), cytokines, and chemokines [19, 20]. Neutrophils can also guide and shape the adaptive immune response, in which T-cell proliferation is regulated by their production of several cytokines and growth factors [21]. T-cell suppression can also be mediated by extracellular arginase and reactive oxygen species, which are produced by neutrophils. Neutrophils also alter the inflammatory environment by producing L-arginine, arginase-1, and large amounts of reactive oxygen species. He et al. reported that peritumoral neutrophils up-regulate Programmed cell death ligand-1 (PDL-1) expression and suppress T-cell proliferation in hepatocellular carcinoma [10, 11], while Wang et al. have reported that tumor-derived Granulocyte macrophage colony-stimulating factor (GM-CSF) activates neutrophils and induces neutrophil PD-L1 expression through the Janus kinase and signal transducer and activator of transcription 3 (JAK-STAT3) pathway [11]. Other researchers have demonstrated that intratumoral neutrophils have prognostic value in cases of renal cell carcinoma, colorectal cancer, and non-small cell lung cancer [22–24], and we have reported that neutrophil infiltration is associated with lymph node micrometastasis and intranodal lymphangiogenesis [3]. In this study, we wanted to show the correlation of intratumoral neutrophils between primary tumor and TDLNs, and our results indicate that TANs in the primary tumor might spread through lymphatic vessels and become involved in cancer-related lymphangiogenesis.

Furthermore, we evaluated whether CD15^+^TANs expressed CXCR2, which is a chemokine receptor that binds to C-X-C motif chemokine ligand (CXCL)1, CXCL3, CXCL5, and CXCL7. This receptor can regulate the recruitment and activation of neutrophils, and possibly mediate the inflammatory response in the tumor microenvironment [25, 26]. Previous studies have confirmed that CXCR2^+^neutrophils are mainly observed in the stroma, where they play critical roles in mediating angiogenic activity, lymph node metastasis, and tumour proliferation in colon cancer, oral squamous cell cancer, oesophageal cancer, and breast cancer [26–29]. In addition, CXCR2+ neutrophils induce the migration of myeloid-derived suppressor cells into tumours [29]. Similarly, we observed that the numbers of CXCR2^+^cells and TANs were correlated in the primary tumors, which indicates that TANs might have similar properties to those of myeloid-derived suppressor cells.

Many investigators have reported that the prognosis of patients with cancer can be predicted using systemic inflammatory indexes, including the NLR, the platelet-lymphocyte ratio, and the Glasgow prognostic score [13, 14, 30, 31]. Among these indexes, NLR is believed to best reflect the host’s immune status and the inflammatory response [13, 32]. The present study also revealed a positive correlation between the pre-operative NLR and the number of CD15^+^TANs in the primary tumor, and the average NLR value increased with tumor progression. These results suggest that excessive neutrophil infiltration into the tumor might mobilize neutrophils from the bone marrow into the peripheral blood, and that local inflammation caused by CD15^+^TANs may be correlated with systemic inflammation in patients with gastric cancer.

The present study has several limitations. First, we only considered a small number of cases, and the cellular interactions between the tumor cells and CD15^+^TANs were not directly observed using an experimental model. Thus, an in vitro experiment will be needed to evaluate the immune regulation that is performed by TANs. Second, we did not determine the neutrophils’ differentiation cluster(s), such as CD66b, CD16b, CD32, and MPO [31, 33–36]. Third, we could not determine how CD15^+^TANs cause tumor progression, induce lymph node metastasis, or spread throughout the body.

## Conclusion

We found that patients with high CD15^+^neutrophil infiltration in gastric cancer tissues had a poorer prognosis, compared to patients with low infiltration. In addition, the number of intratumoral neutrophils was associated with the systemic inflammatory response. Therefore, our findings suggest the TANs might play an important role in the induction of inflammation, which might allow gastric carcinoma cells to spread and induce lymph node metastasis.

## Abbreviations

CXCL: C-X-C motif chemokine ligand
CXCR2: C-X-C motif chemokine receptor 2
EMT: Epithelial-Mesenchymal Transition
FBS: Fetal bovine serum
GM**-**CSF: Granulocyte macrophage colony-stimulating factor
IL-6: interleukin 6
JAK-STAT3: Janus kinase and signal transducer and activator of transcription 3
MMP: Matrix metalloproteinase
MPO: Myeloperoxidase
NLR: Neutrophil-lymphocyte ratio
PBS: Phosphate-buffered saline
PDL-1: Programmed cell death ligand-1
TANs: Tumor-associated neutrophils
TDLNs: Tumor-draining lymph nodes
VEGF: Vascular endothelial growth factor
